# A CLDN1-Negative Phenotype Predicts Poor Prognosis in Triple-Negative Breast Cancer

**DOI:** 10.1371/journal.pone.0112765

**Published:** 2014-11-13

**Authors:** Fei Ma, Xiaoyan Ding, Ying Fan, Jianming Ying, Shan Zheng, Ning Lu, Binghe Xu

**Affiliations:** 1 Department of Medical Oncology, Cancer Hospital, Chinese Academy of Medical Sciences and Peking Union Medical College, Beijing, China; 2 Department of Medical Oncology, Beijing DiTan Hospital, Capital Medical University, Beijing, China; 3 Department of Pathology, Cancer Hospital, Chinese Academy of Medical Sciences and Peking Union Medical College, Beijing, China; Rajiv Gandhi Centre for Biotechnology, India

## Abstract

**Introduction:**

Triple-negative breast cancer (TNBC) is a heterogeneous disease with no definitive prognostic markers. As a major component of tight junctions, claudins (CLDNs) presumably play an important role in carcinogenesis and progression of breast cancer. This study was aimed at determining the relationship between the expression of CLDNs and the clinical outcomes of TNBCs.

**Materials and Methods:**

The surgical specimens of primary breast tumors from a consecutive cohort of 173 TNBC patients were retrospectively collected. The membranous expression of CLDN1, CLDN2, CLDN4, and CLDN7 was measured by immunohistochemistry. Then, the associations between CLDN expression, clinicopathological features, and clinical outcomes were assessed.

**Results:**

Positive CLDN1, CLDN2, CLDN4, and CLDN7 membrane expression was detected in 44.5%, 54.9%, 76.9%, and 73.4% of the cohort specimens, respectively. A lack of CLDN1 expression was related to only lymph node metastasis (P = 0.014). The rate of CLDN4-positive tumors was significantly increased in tumors of a higher grade (P = 0.003). Importantly, negative CLDN1 expression was associated with worse relapse-free survival (RFS) in both lymph node positive (LN+) and negative (LN−) cases (both P<0.001). Similarly it was also associated with shorter overall survival (OS)(P = 0.003 in LN+ cases; P = 0.018 in LN− cases). In the LN+ subgroup, CLDN2-negative cases had a significantly higher risk of recurrence (P = 0.008). Multivariate analysis revealed that negative CLDN1 expression was an independent prognostic factor for high risk of both recurrence and death (HR 5.529, 95% CI 2.664–11.475, P<0.001; HR 3.459, 95% CI 1.555–7.696, P = 0.002). However, neither CLDN4 nor CLDN7 expression was associated with survival.

**Conclusion:**

In TNBC, the CLDN1-negative phenotype predicts a high risk of recurrence and death. The absence of CLDN1 expression is strongly suggested to be an independent adverse prognostic factor in this heterogeneous subtype of breast cancer.

## Introduction

Triple-negative breast cancer (TNBC) is a therapeutically relevant definition of a subgroup of breast cancers (BCs) characterized by the absence of staining for the estrogen receptor (ER), the progesterone receptor (PR), and human epidermal factor receptor 2 (HER2). TNBC has proven to be remarkably heterogeneous with various prognoses stratified by clinical, pathological, genetic factors, and treatment modalities [1]–[5]. For example, stage II TNBC cases treated with adjuvant chemotherapy would have a better prognosis than those receiving no chemotherapy [5]. Certain histological types, such as metaplastic carcinomas, have been shown to have a very poor outcome, but medullary carcinomas have been shown to have a particularly good prognosis [6]. In addition, the “basal-like” type (with CK5/6 and EGFR expression) of TNBC generally has a worse prognosis than non-basal-like TNBC [7], [8]. Due to a lack of specific targets for treatment, standard chemotherapy regimens for TNBC have not been established and dose-dense chemotherapy regimens tend to be effective in improving survival [9], [10]. Thus far, data on definitive predictive markers of TNBC are insufficient. Therefore, it is urgent that we elucidate novel predictive biomarkers of TNBC to assist in selecting patients with high-risk tumors before initiating dose-dense chemotherapy to avoid overtreatment-related complications and to identify potential molecular targets.

Claudins (CLDNs), a family comprising 27 members, are the primary family of proteins that make up tight junctions between neighboring cells [11]. As major trans-membrane proteins, CLDNs play crucial roles in the formation and maintenance of tight junctions [12]. It is generally accepted that the disruption of tight junctions leads to the loss of intercellular cohesion, which contributes to the invasiveness and lack of differentiation of cancer cells and thus promotes metastasis. Earlier studies have suggested that mRNA or membrane protein expression levels of CLDNs were strongly correlated with carcinogenesis in BC and especially CLDN1 [13]–[19]. However, clinical studies are still relatively limited. Two reports have suggested a correlation between CLDN1 down-regulation and BC recurrence [16], [17]. One study reported that down-regulation of CLDN2 was associated with advanced BC [18]. Of the few publications on CLDN7 expression in BC, researchers have reported that positive CLDN7 expression was significantly associated with an increased risk of recurrence and nodal involvement but with lower histological grade in a small sample of invasive ductal carcinoma (IDC) tumors [19], [20]. The roles of the four CLDNs listed above are not well understood with respect to the various subtypes of TNBC.

Recently, a claudin-low phenotype of BC was described as a new subtype by gene microarray. It is typically triple negative by immunohistochemistry (IHC) and accounts for 25–39% of TNBCs. Defined by low mRNA expression of CLDN3, CLDN4, and CLDN7 [21], this subtype was reported to be a frequent phenomenon in metaplastic and basal-like BCs and has been shown to have a poor prognosis similar to that of basal-like BCs [21]–[23]. However, the expression profiles of CLDNs in TNBC have not yet been well analyzed.

Taken together, we hypothesize that the expression of CLDNs, including CLDN1, CLDN2, CLDN4, and CLDN7, associates with prognostic heterogeneity of TNBC. Therefore, we associated clinicopathological parameters with protein expression of CLDN1, CLDN2, CLDN4, and CLDN7 to uncover prognostic biomarkers for TNBC.

## Materials and Methods

### Ethics statement

This study was a retrospective study. All of the specimens were retrieved from the Biological Specimen Bank. The study was approved by the Independent Ethics Committee of the Cancer Hospital, Chinese Academy of Medical Sciences (CH-BC-018). The informed consent was also remitted by the Ethics Committee.

### Study population

Between June 1, 2004 and January 1, 2007, a total of 2835 operable BC patients at the Cancer Hospital, Chinese Academy of Medical Sciences (CAMS) were retrospectively collected for this study. Of these, 292 patients were identified as TNBC cases. Triple-negative breast cancer was defined as estrogen receptor/progesterone receptor <1% [24] and HER2 0, 1+ or 2+ (with negative fluorescence by in situ hybridization) on immunohistochemistry. A gene copy/CEP-17 ratio <2.0 was considered to indicate negative amplification [25]. A total of 119 patients were excluded from the analysis because they had synchronous or metachronous bilateral BCs (n = 4), pure ductal carcinoma in situ (n = 4), other malignant tumors (n = 8), their tumor specimens were not archived properly (n = 71), or they missed a follow-up (n = 32). In total, 173 cases were included, and the surgical specimens of the primary breast tumors prior to adjuvant chemotherapy were retrieved from the Biological Specimen Bank of the Cancer Hospital, CAMS. Data on the patients’ medical history, tumor features, demographic characteristics, and treatment modalities were recorded. Staging of tumors was performed according to the American Joint Committee on Cancer Staging Group’s Cancer Staging Manual [26]. Grading and histologic classification of the tumors were based upon the WHO’s criteria [27]. All of the patients undergoing breast-conserving surgery received post-operative radiotherapy. Adjuvant chemotherapy was administered depending on the risk of recurrence in accordance with the National Comprehensive Cancer Network guidelines [28].

### Immunohistochemistry (IHC)

To measure CLDN expression, polyclonal antibodies against claudin-1 (1∶50) and monoclonal antibodies against claudin-2 (12H12, 1∶100), claudin-4 (3E2C1, 1∶50), and claudin-7 (5D10F3, 1∶50) were used. The four CLDN antibodies were purchased from Zymed (CA, USA). The primary antibodies were detected using a secondary antibody conjugated to HRP (Cytomation Envision System HRP, DAKO, Carpinteria, CA). Diaminobenzidine was used as a chromogen. Sections were counterstained with hematoxylin. Normal breast skin served as a positive control for CLDN1 and CLDN2, and normal colon tissue served as a positive control for CLDN4 and CLDN7. In negative control slides, the same method was employed and the primary antibody was substituted with 1% TBS.

### Interpretation of IHC sections

Based on previous studies, CLDN1, CLDN2, CLDN4 and CLDN7 IHC staining was interpreted according to the extent of membrane staining [16], [22], [29]. Two independent pathologists observed the immunostaining under a light microscope at a 200× magnification, and positive cells, negative cells and total cells from five different visual fields were counted for each specimen. For pathological results with discrepancies, a third pathologist was asked to independently examine the slides to reach a unanimous decision. Scoring was performed as follows: negative (**−**), <10% positive tumor cells; positive (+), **≥**10% positive tumor cells [16]. Only membranous staining was considered positive staining [29]. Furthermore, interstitial lymphocyte infiltration (ILI) was defined as the number of lymphocytes in the tumor interstitium in five different visual fields.

### Statistical analysis

Statistical analysis was performed using SPSS 16.0 statistical software (SPSS Inc, Chicago, IL, USA). Relapse-free survival (RFS) was measured from the date of curative surgery to the first day of documented recurrence. Overall survival (OS) was measured from the date of curative surgery to the date of death or final follow-up.

Independent sample t-tests and chi-square tests were used to compare continuous and categorical variables. The Kaplan-Meier product limit method was used to estimate the survival outcomes; groups were compared using the log-rank test. Cox proportional hazards models were fit to determine the association between clinicopathological characteristics, especially CLDN expression, and patient survival. P<0.05 was considered to be statistically significant.

## Results

### Baseline clinicopathological characteristics by lymph node status

The study cohort had a median age of 54 years (range: 24–78 years). The median follow-up time for the cohort was 64.6 months (range: 8.1–95.8 months). Table 1 lists the demographics, characteristics, treatment, and metastatic patterns of the cohort according to nodal status. According to the status of lymph node metastasis, patients were stratified into two subgroups: node positive (LN+, n = 97) and node negative (LN−, n = 76). Patients in the LN+ subgroup tended to have a more advanced stage of disease and a larger tumor size with more vascular involvement (P<0.001) than those in the LN− group. Among all patients, 161 (93.1%) received adjuvant chemotherapy, consisting of primarily anthracycline-and/or taxane-based regimens. At the end of follow-up, 67 patients had relapsed and 47 patients had died from metastatic BC. Although a significantly larger number of patients in the LN+ group received radiotherapy, more patients relapsed in the LN+ group than in the LN− group (P<0.001). The sites of metastasis were similar between the LN+ and LN− subgroups.

**Table 1:** Demographics and characteristics of patients by nodal status.

Clinical characteristics	Nodal positive (n = 97) n (%)	Nodal negative (n = 76) n (%)	P value
Age (mean±SD)	50.1±11.9	52.4±11.1	0.197^a^
Body weight index (mean±SD)	25.0±3.6	25.0±3.6	0.948 ^a^
Family history	17 (17.5)	16 (21.1)	0.565
Histologic type			0.389
IDC	92 (94.9)	73 (96.1)	
Metaplastic	3 (3.1)	0 (0)	
Medullary	1 (1.0)	2 (2.6)	
ILC	1 (1.0)	1 (1.3)	
Tumor grade			0.541
Grade 2	47 (48.5)	33 (43.4)	
Grade 3	50 (51.5)	43 (56.6)	
Tumor size (>2 cm)			0.01
T1	24 (24.7)	34 (44.7)	
T2/T3/T4	73 (75.3)	42 (55.3)	
Stage			<0.001
I	0 (0)	33 (43.4)	
II	39 (40.2)	43 (56.6)	
III	58 (59.8)	0(0)	
Vascular involvement	27 (27.8)	4 (52.6)	<0.001
Surgery Mode			0.153
Modified Radical Mastectomy	89 (91.7)	64 (88.2)	
Breast conservation	8 (8.3)	12 (11.2)	
Adjuvant radiotherapy	59 (60.8)	16 (21.1)	<0.001
Adjuvant chemotherapy	93 (95.9)	68 (89.5)	0.133
Adjuvant Taxane	68(73.1)	32(47.1)	0.001
Adjuvant Anthracycline	92(98.9)	66(97.1)	0.574
Recurrence	51 (52.6)	16 (21.1)	<0.001
Site of recurrence before death			
Local-regional	10 (19.6)	7 (43.8)	0.096
Non-visceral	36 (70.6)	11 (68.8)	1.0
Visceral	43 (84.3)	13 (81.3)	0.716

%: positive numbers/total numbers of the subgroup according to nodal status; IDC: infiltrating ductal carcinoma; ILC: infiltrating lobular carcinoma; ^a^ by *t* test; other data were evaluated by *X* ^2^ test;”recurrence” was defined before the last follow-up.

### Expression of CLDN1, CLDN2, CLDN4, and CLDN7

Membrane expression of CLDN1, CLDN2, CLDN4, and CLDN7 was detected in 44.5% (77), 54.9% (95), 76.9% (133), and 73.4% (127) of the patients, respectively. Only one patient was positive for CLDN1, CLDN2, and CLDN7 expression, and two of three patients with medullary carcinomas were positive for CLDN4 expression. For three cases with metaplastic tumors, positive expression of CLDN1, CLDN2, CLDN4, and CLDN7 was detected in 0, 1, 3, and 2 cases, respectively. Both CLDN2 and CLDN4 expression associated with positive expression of CLDN1 with p values <0.001 and = 0.014, respectively. Similarly, CLDN7 expression was associated with CLDN4 expression (P = 0.001) (Table S1).

**Figure 1:**
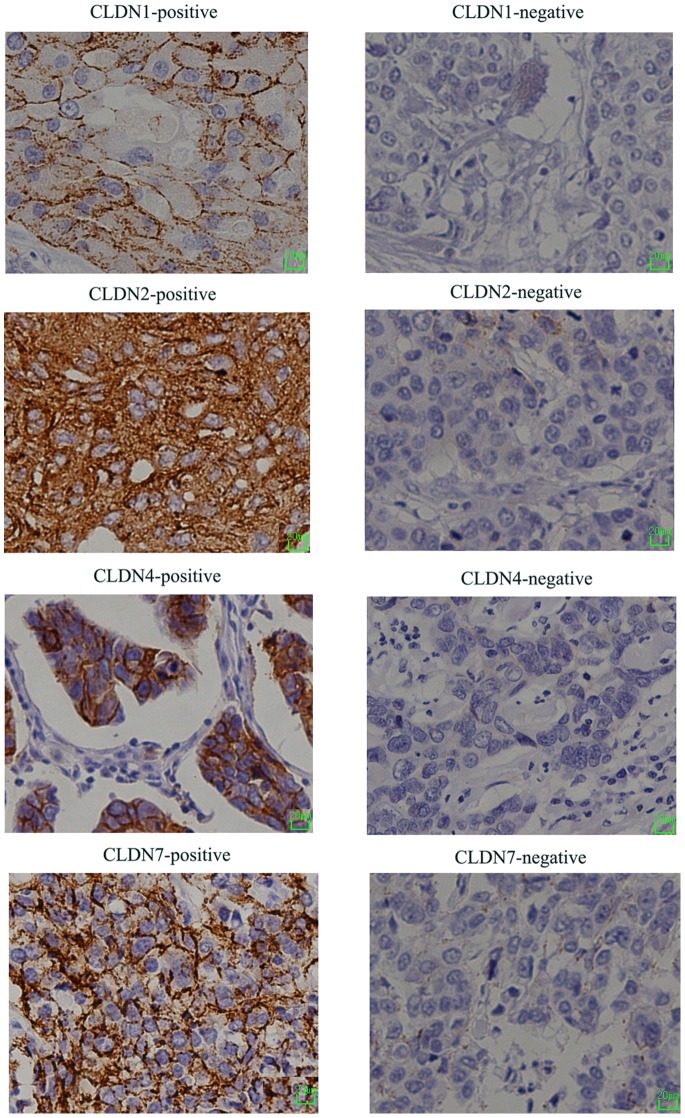
Membrane protein expression was assessed in 173 TNBC specimens using our optimized CLDNs IHC protocol. Representative CLDN-positive and CLDN-negative sections are shown. CLDN expression was primarily localized to the membrane (≥10% tumor cells), and CLDN2 was also expressed in the cytoplasm. (Original magnification: ×200.) Cells nuclei were counterstained with hematoxylin.

Expression of the four CLDNs was predominantly localized to the membrane, but CLDN2 was also expressed in the cytoplasm of some TNBC specimens (Fig. 1). CLDN1 and CLDN2 stained positive in 10–50% of tumor cells, while CLDN4 and CLDN7 stained positive in ≥50% of tumor cells.

### Association between CLDN expression and clinical parameters

Chi-square tests were used to compare CLDNs expression in the LN− and LN+ groups. CLDN1 expression was significantly higher in the LN− group, 55.3% (42/76) compared to 36.1% (35/97) in the LN+ group, suggesting that the absence of CLDN1 expression is related to lymph node metastasis (P = 0.014) (Table 2). CLDN1 expression was also associated with interstitial lymphocyte infiltration (P = 0.017) (Table 2). No significant differences were observed between the subgroups stratified by other clinicopathological parameters (age, histology grade, vascular involvement, and tumor size) and CLDNs expression, except CLDN4 expression was found to be significantly higher in TNBC patients ≤50 years old with a tumor grade 3 compared to TNBC patients >50 years old or having a tumor grade 2 (P = 0.012 and P = 0.003, respectively) (Table 2).

**Table 2:** Comparison of CLDN expression and clinical parameters.

Clinical parameters	CDLN1+ % (n)	CLDN2+ % (n)	CLDN4+ % (n)	CLDN7+ % (n)
Nodal status				
Negative (n = 76)	55.3%(42)^a^	60.5% (46)	76.3% (58)	77.6% (59)
Positive (n = 97)	36.1% (35)^a^	50.5% (49)	77.3% (75)	70.1% (68)
Age				
≤50 years (n = 87)	42.5% (37)	55.2% (48)	85.1% (74)^b^	67.8% (59)
>50 years (n = 86)	46.5% (40)	54.7% (47)	68.6% (59)^b^	79.1% (68)
Vascular involvement				
Yes (n = 31)	35.5% (11)	61.3% (19)	80.6% (25)	74.2%(23)
No (n = 142)	46.5% (66)	53.5% (76)	76.1% (108)	73.2% (104)
Tumor size				
≤2 cm (n = 59)	47.5% (28)	54.2% (32)	78.0% (46)	72.9% (43)
>2 cm (n = 114)	43.0% (49)	55.3% (63)	76.3% (87)	73.7% (84)
Histology				
IDC (n = 167)	45.5% (76)	55.7% (93)	76.6% (128)	74.3% (124)
MC (n = 6)	16.7% (1)	33.3% (2)	83.3% (5)	50.0% (3)
ILI				
Yes (n = 31)	64.5% (20)^c^	64.5% (20)	80.6% (25)	74.2% (23)
No (n = 142)	40.1% (57)^c^	52.8% (75)	76.1% (108)	73.2% (104)
Tumor grade				
Grade 2 (n = 72)	38.9% (28)	55.6% (40)	65.3% (47)^d^	77.8% (56)
Grade 3 (n = 93)	50.5% (47)	55.9% (52)	86.0% (80)^ d^	69.9% (65)

%: number of positivity/total number of the subgroup; MC, included 3 medullary carcinomas and 3 metaplastic carcinomas; IDC: invasive ductal carcinoma; ILI: interstitial lymphocyte infiltration. ^a^ P = 0.014,^ b^P = 0.012, ^c^P = 0.017, ^d^P = 0.003.

### Significance of CLDN expression in TNBC

**Figure 2:**
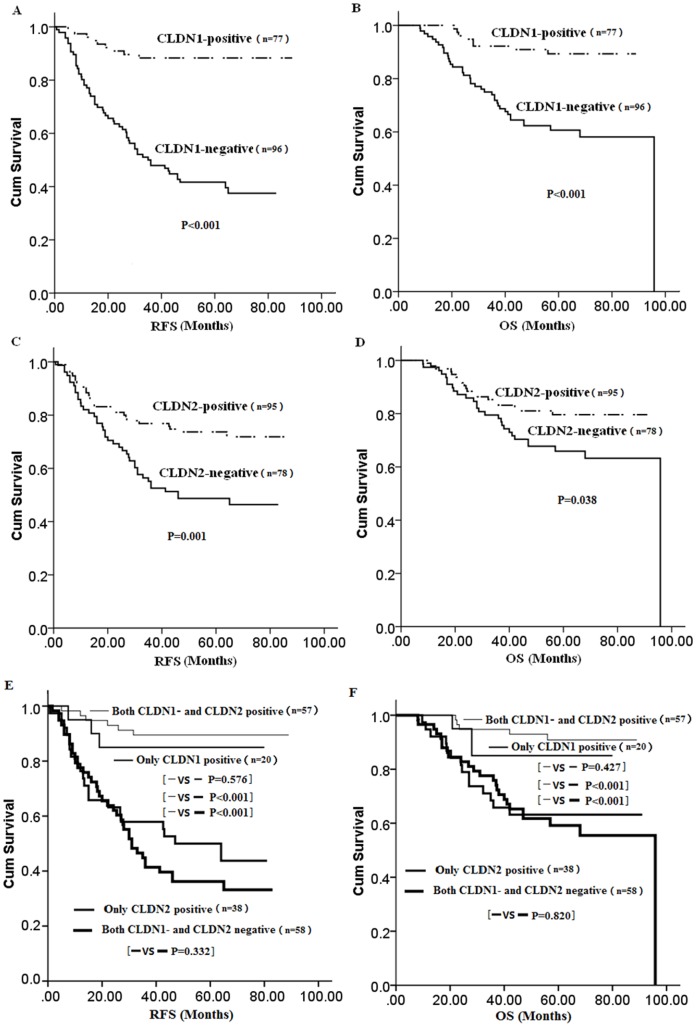
Kaplan-Meier survival curves of RFS and OS based on CLDN1- and CLDN2–membrane expression (CLDN1: A for RFS, B for OS; CLDN2: C for RFS, D for OS); (combination of CLDN1 and CD LN−2: E for RFS, F for OS).

The 5-year RFS and OS rates of 173 TNBC were 61.4% and 73.5%, respectively. All clinical factors were investigated by univariate analysis to determine whether there was a significant difference in RFS (Table 3). Among all TNBC patients, negative CLDN1 or CLDN2 expression was associated with significantly worse RFS (P<0.001 and P = 0.001, respectively) (Fig. 2A, 2C). Other factors such as node positive, vascular invasion, and with adjuvant radiotherapy were also related to a high risk of recurrence(P<0.001, 0.027 and 0.014, respectively) (Table 3). Similar associations were noted between CLDN1, CLDN2 expression and OS (P<0.001 and P = 0.038, respectively) (Fig. 2B, 2D).

**Table 3:** Univariate analyses of RFS by clinical factors.

Clinical factors	Events (n, %)	HR (95% CI)	P value
CLDN1 expression		0.139 (0.069–0.281)	<0.001
Negative	58 (60.4%)		
Positive	9 (11.7%)		
CLDN2 expression		0.450 (0.275–0.737)	0.001
Negative	41 (52.6%)		
Positive	26 (27.4%)		
CLDN4 expression		1.362 (0.743–2.496)	0.317
Negative	13 (32.5%)		
Positive	54 (40.6%)		
CLDN7 expression		0.764 (0.453–1.289)	0.313
Negative	20 (43.5%)		
Positive	47 (37.0%)		
Age		0.786 (0.486–1.271)	0.327
≤50 years	36 (41.4%)		
>50 years	31 (36.0%)		
BMI (Kg/m^2^)		1.170 (0.725–1.889)	0.520
≤25	33 (36.3%)		
>25	34 (41.5%)		
Nodal status		3.276 (1.866–5.752)	<0.001
Negative	16 (21.1%)		
Positive	51 (52.6%)		
Tumor size		1.600 (0.932–2.747)	0.088
<2 cm	18 (30.5%)		
>2 cm	49 (43.0%)		
Tumor Grade		0.717(0.439–1.170)	0.183
Grade 2	33 (45.8%)		
Grade 3	31 (33.3%)		
With vascular invasion		1.864 (1.074–3.235)	0.027
No	50 (35.2%)		
Yes	17 (54.8%)		
Adjuvant radiotherapy		1.822 (1.127–2.948)	0.014
No	31 (31.6%)		
Yes	36 (48.0%)		
(Neo) adjuvant chemotherapy		0.739 (0.319–1.710)	0.480
No	6 (50.0%)		
Yes	61 (37.9%)		
Chemo with taxanes		0.903 (0.542–1.505)	0.696
No	25 (41.0%)		
Yes	36 (36.0%)		

%: number of events/total number of the subgroup.

Further analysis revealed that cases with both CLDN1- and CLDN2-positive expression had a similar survival rate as cases with only positive CLDN1 expression (P = 0.576 for RFS and P = 0.427 for OS), but these cases had significantly longer RFS and OS than those cases that were positive for only CLDN2 expression or those cases that were negative for CLDN1 and CLDN2 expression (P<0.001 for each comparison) (Fig. 2E, 2F). Importantly, negative CLDN1 expression was associated with worse relapse-free survival (RFS) in both lymph node positive (LN+) and negative (LN−) cases (both P<0.001). Similarly it was also associated with shorter overall survival (OS)(P = 0.003 in LN+ cases; P = 0.018 in LN− cases). However, negative CLDN2 expression was associated with worse RFS in only LN+ cases, P = 0.008 (Fig. S1).

Similar results were observed for the 161 patients who received surgery plus adjuvant chemotherapy as for the whole cohort of TNBC cases. Both negative CLDN1 and CLDN2 expression were associated with significantly worse survival in terms of RFS and OS (RFS, P<0.001 and P = 0.001, respectively; OS, P<0.001 and P = 0.035, respectively) (Fig. S2).

Multivariate Cox regression analysis for RFS identified negative CLDN1 expression to be an independent adverse factor for tumor recurrence and death (HR 5.529, 95% CI 2.664–11.475, P<0.001; HR 3.459, 95% CI 1.555–7.696, P = 0.002) (Table 4, 5). Lymph node metastasis was also associated with RFS and OS in the multivariate analysis (P = 0.005 and P = 0.004, respectively). No other clinical factors were associated with a significantly higher risk of recurrence or death in the multivariate analysis (Table 4, 5).

**Table 4:** Multivariate Cox regression analysis of RFS.

Factors	HR (95% CI)	P value
CLDN1 negativity	5.529 (2.664–11.475)	<0.001
Lymph node metastasis	2.339 (1.292–4.233)	0.005
CLDN2 negativity	1.424 (0.854–2.373)	0.175
Adjuvant radiotherapy	1.150 (0.670–1.974)	0.613
With vascular invasion	1.289 (0.698–2.381)	0.417
Tumor size >2 cm	1.293 (0.739–2.264)	0.368

**Table 5:** Multivariate Cox regression analysis of OS.

Factors	HR (95% CI)	P value
CLDN1 negativity	3.459 (1.555–7.696)	0.002
Lymph node metastasis	3.496 (1.500–8.150)	0.004
CLDN2 negativity	1.310 (0.708–1.425)	0.390
Adjuvant radiotherapy	1.557 (0.795–3.050)	0.196
With vascular invasion	1.394 (0.695–2.795)	0.350
Tumor size >2 cm	1.495 (0.726–3.078)	0.275

## Discussion

TNBC has already been widely acknowledged as a distinct subtype of BC associated with a poor prognosis [2], [3]. Increasing evidence has shown that TNBC is a heterogeneous group of diseases with different biological characteristics and clinical outcomes [2], [3]. Claudins (CLDNs), major components of tight junctions, presumably play an important role in carcinogenesis and progression of BC, but little is known about the impact of these molecules on tumor recurrence. Furthermore, “claudin-low” is a special subtype of BC identified by gene microarray that is associated with a poor prognosis. Most patients with “claudin-low” are triple negative. But the associations between the CLDNs-negative phenotypes, defined by immunohistochemical methods, and the “claudin-low” subtype were unclear. Before this study, it was not sure whether the CLDNs-negative phenotypes associated with the poor prognosis in TNBC.

In accordance with previous studies, positive CLDN expression was defined as ≥10% membrane expression for four CLDNs in this study [12], [16]. Positive CLDN1 and CLDN2 expression was detected in 44.5% and 55.9% of cases, respectively. And the extent of immunostaining in most cases was 10–50%. Consistent with our results, Marohashi’s study showed that CLDN1 was expressed in 61.4% of 83 BCs (cut-off value 10%) [16]. Because a different criterion for positive expression was used, the rate of CLDN1-high expression was much lower in Gerhard’s study, with only 12.6% in 103 TNBC tumors having high levels of CLDN1 expression [23]. In addition, the majority of TNBC patients showed positive membrane staining of CLDN4 (76.9%) and CLDN7 (73.4%). Similar to our results, increased protein expression of CLDN4 was found in ER-negative BC, especially in basal-like BC (in which subtype tumors were mainly TNBC) [22], [30], [31]. However, a study conducted by Blanchard et al. found contradictory results in 152 breast tumors. No significant difference in CLDN4 expression was observed between basal-like BC and non-basal-like BC (P = 0.18) [32]. Therefore, further investigations in CLDN4 expression levels and TNBC subtypes should be performed.

In this study, positive CLDN1 expression was associated with interstitial lymphocyte infiltration. These lymphocytes may down-regulate cytokines to induce the expression of CLDN1. For instance, loss of keratin 8 and 18 expression could increase CLDN1 expression in epithelial cancer cells [33]. However, negative CLDN1 expression was significantly more frequent in the LN+ TNBC subgroup compared to the LN− subgroup (55.3% vs. 36.1%, P = 0.014). These finding were consistent with one study that demonstrated that CLDN1-negative expression was associated with node metastasis in 83 BCs [16]. Thus, loss of CLDN1 may lead to lack of intercellular cohesion between cancer cells, promote invasiveness, and contribute to lymph node metastasis [15]. This finding suggests that CLDN1 may play a pivotal role in the invasion of TNBC. In addition, positive CLDN4 expression was associated with higher tumor grade (P = 0.003). In addition, a study conducted by Szasz et al. with 97 cases of IDC and invasive lobular carcinomas reported a similar result [29]. In agreement with previous studies of TNBCs [2], [3], patients with node metastasis had a significantly worse prognosis in terms of RFS (P<0.001).

More importantly, this study consisted of the largest series of TNBCs to clarify the relationship between CLDN1 expression and survival outcomes. We demonstrated that a lack of CLDN1 expression on the membrane was associated with worse RFS and OS in both LN+ and LN− cases. One mechanism to explain this phenomenon could be that, as in the case of E-cadherin, the transcription factors slug and snail, which are key markers of EMT, could bind to the *CLDN1* promoter to repress its activation and promote the activation of *MMP-2* and *MMP-9*, resulting in TNBC invasion [34], [35]. In line with our observations, studies conducted by Morohashi et al. and Szasz et al. with a smaller sample of BCs reported that CLDN1-negative or -low expression was associated with tumor recurrence in different subtypes of BC (P<0.001 and P = 0.038) [16], [29]. In contrast, research conducted by Kolokytha et al. with 76 TNBC tumors showed that CLDN1 expression was not related to survival [36]. In the 161 TNBC cases that received adjuvant chemotherapy, negative CLDN1 expression was also associated with a worse outcome (P<0.001). Additionally, CLDN1-negative expression was identified as an adverse prognostic factor by multivariate Cox regression analysis. These results suggest that negative CLDN1 expression may be an adverse prognostic factor in TNBC.

In the current study, in subgroups analysis by lymph node status, we also observed that CLDN2-negative expression was only associated with worse RFS in LN+ TNBC cases (P = 0.008). In a previous study, Kim reported that down-regulation of CLDN2 in breast carcinomas was related to advanced disease and lymph node metastasis [18]. Tabariès et al. identified that only decreased expression of CLDN2 promoted breast cancer liver metastases by reducing adhesion between tumor cells [37]. Our results further supported previous studies that imply that CLDN2 is implicated in the development of metastatic potential within TNBC.

Finally, neither CLDN4 nor CLDN7 expression was significantly associated with survival of patients with TNBC, which contradicts the previously reported association between CLDN4 expression and BC tumor recurrence [29], [36]. A Japanese group demonstrated that there was no association between CLDN4 expression and BC tumor recurrence [16]. However, IHC analysis performed on tissue microarray samples from 97 BC patients by Szasz et al. demonstrated that higher expression of CLDN4 was significantly associated with increased risk of recurrence (P = 0.045) [29]. Conversely, Kolokytha et al. analyzed 76 TNBC tumors and found that positive CLDN4 expression was associated with a favorable prognosis [36]. Further investigation of a larger sample of TNBCs is needed.

Because this was a retrospective study, a few limitations were expected. First, the sample size determination was not previously planned, and the detrimental effects of a possibly underpowered study on some significant associations between CLDNs and clinical outcomes could be unavoidable. Second, no validation cohort was used to confirm the positive or negative findings.

## Supporting Information

Figure S1 **Kaplan-Meier survival curves of RFS and OS based on CLDN1 and CLDN2 membrane expression by lymph node status (LN− subgroup: CLDN1, A for RFS and C for OS, CLDN2, E for RFS, G for OS; LN+ subgroup: CLDN1, B for RFS and D for OS, CLDN2, F for RFS, H for OS).**
(TIF)

Figure S2 **Kaplan-Meier survival curves of RFS and OS in the (neo) adjuvant chemotherapy group (RFS: A for CLDN1, B for CLDN2; OS: C for CLDN1, D for CLDN2).**
(TIF)

Table S1 **The associations between the 4 CLDNs expression.**
(DOC)
