# Supplementary figures and images for: A CLDN1-Negative Phenotype Predicts Poor Prognosis in Triple-Negative Breast Cancer
Source: PLoS One. 2014 Nov 13;9(11):e112765. doi: 10.1371/journal.pone.0112765 (PMC4231092; doi:10.1371/journal.pone.0112765)

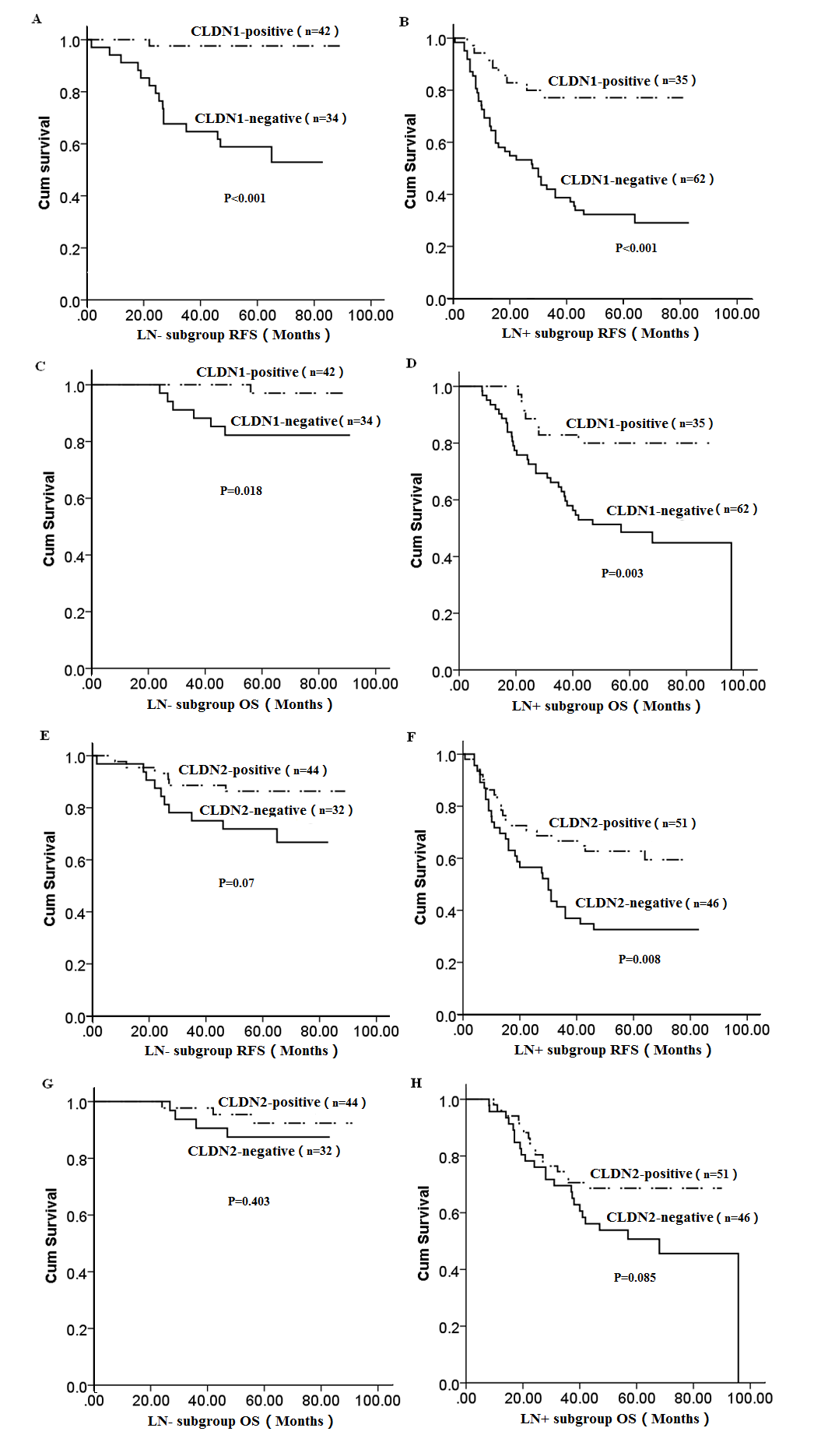

Supplement: Figure S1 — Kaplan-Meier survival curves of RFS and OS based on CLDN1 and CLDN2 membrane expression by lymph node status (LN− subgroup: CLDN1, A for RFS and C for OS, CLDN2, E for RFS, G for OS; LN+ subgroup: CLDN1, B for RFS and D for OS, CLDN2, F for RFS, H for OS). (TIF) [file pone.0112765.s001.tif]

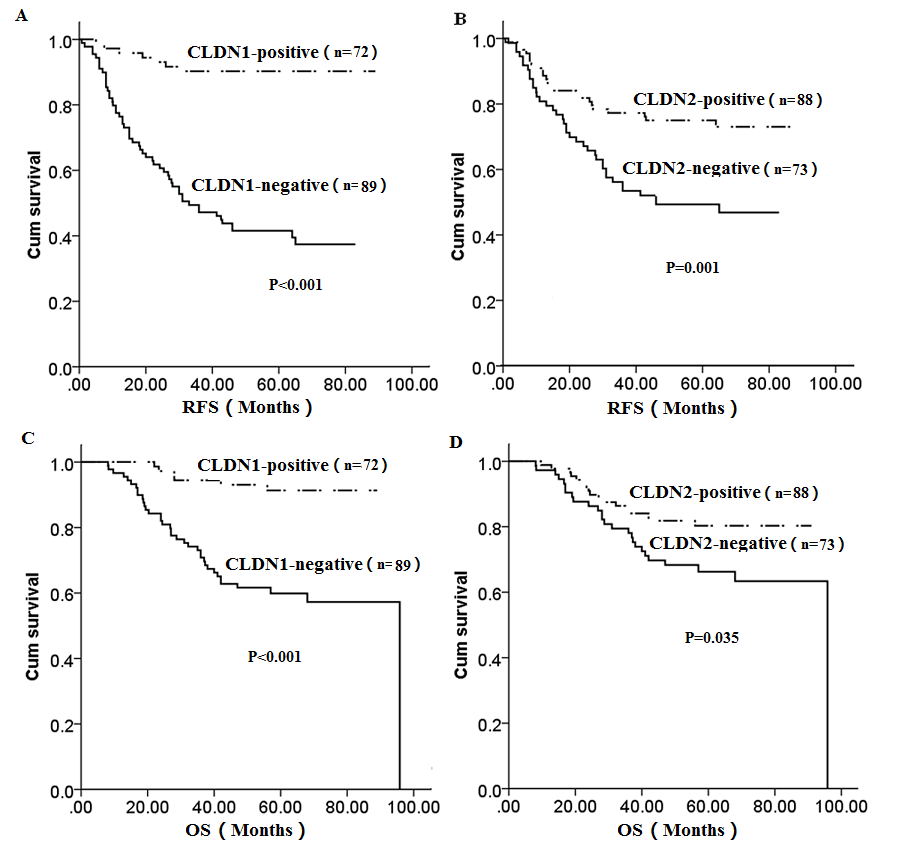

Supplement: Figure S2 — Kaplan-Meier survival curves of RFS and OS in the (neo) adjuvant chemotherapy group (RFS: A for CLDN1, B for CLDN2; OS: C for CLDN1, D for CLDN2). (TIF) [file pone.0112765.s002.tif]
