# Supplementary material for: A CLDN1-Negative Phenotype Predicts Poor Prognosis in Triple-Negative Breast Cancer
Source: PLoS One. 2014 Nov 13;9(11):e112765. doi: 10.1371/journal.pone.0112765 (PMC4231092; doi:10.1371/journal.pone.0112765)
Supplement: Table S1 — The associations between the 4 CLDNs expression. (DOC) [file pone.0112765.s003.doc]

Table S1: The associations between the 4 CLDNs expression

| **Factors** | CLDN2  (%, N) | CLDN4  (%, N) | CLDN7  (%, N) |
| --- | --- | --- | --- |
| CDLN1 positivity (n=77) | 74.0% (57)^a^ | 85.7% (66)^b^ | 79.2% (61) |
| CLDN1 negativity (n=96) | 39.6% (38)^a^ | 69.8% (67)^b^ | 68.8% (66) |
| CDLN2 positivity (n=95) | -- | 81.1% (77) | 78.9% (75) |
| CLDN2 negativity (n=78) | -- | 76.9% (60) | 66.7% (52) |
| CDLN4 positivity (n=133) | -- | -- | 79.7% (106)^c^ |
| CLDN4 negativity (n=40) | -- | -- | 52.5% (21)^c^ |

%: number of positivity / total number of the subgroup; ^a^P<0.001, ^b^P=0.014, ^c^P=0.001.
